# The risk of cross-border pollution and the influence of regional climate on the rainwater chemistry in the Southern Carpathians, Romania

**DOI:** 10.1007/s11356-019-07478-9

**Published:** 2020-01-08

**Authors:** Ágnes Keresztesi, Ion-Andrei Nita, Marius-Victor Birsan, Zsolt Bodor, Róbert Szép

**Affiliations:** 1 Faculty of Natural Sciences, Doctoral School of Chemistry, University of Pécs, Ifjúság 6, Pécs, 7624 Hungary; 2 Department of Bioengineering, Sapientia Hungarian University of Transylvania, Piaţa Libertăţii 1, 530104 Miercurea Ciuc, Romania; 3 Institute for Research and Development for Hunting and Mountain Resources, Progresului 35B, 530240 Miercurea Ciuc, Romania; 4 Alexandru Ioan Cuza University, Bulevardul Carol I 11, 700506 Iași, Romania; 5 National Meteorology Administration, Soseaua București-Ploiești 97, District 1, 013686 Bucharest, Romania

**Keywords:** Rainwater chemistry, Ecological risk, Heavy metals, Wet deposition, Long-range transport, Source analysis

## Abstract

The aim of this study is the assessment of rainwater composition, regarding the various sources of major ions and heavy metals, taking into account the characteristic atmospheric circulations and the main air mass transport routes. Rainwater samples were analyzed for pH, electrical conductivity, major ions, and heavy metals. At all sampling sites, the most abundant anions were SO_4_^2ˉ^ and Clˉ, while the dominant cations were Ca^2+^ and Mg^2+^. Regarding heavy metals, the dominance of Pb and Cd was found. The contribution of soil dust from the mining activities and the dissolution of CaCO_3_, MgCO_3_, and CaSO_4_·2H_2_O in the rainwater explains the high concentrations of Ca^2+^, Mg^2+^, and SO_4_^2−^. The overall precipitation contamination with heavy metals at the three sampling sites was assessed by the degree of contamination, showing that Pb and Cd presents the highest risks of all heavy metals. The values of toxicity potential suggested an elevated risk for human health in case of rainwater ingestion, especially in rural areas. Spearman correlation and PCA indicated that the chemical characteristic of the rainwater is primarily controlled by sources such as agricultural activities, mixed and crustal sources, traffic, and other anthropogenic, industrial influences, mining activities, smelting operations, coal combustion, and metal production.

## Introduction

Atmospheric precipitation is one of the most debated subjects worldwide, because of awareness of the impact of acid rain on the environment and human health, but it also provides useful information about the composition of the atmosphere and the importance of different sources of gaseous and particulate pollutants. Characteristics of rainwater chemistry in urban areas can be attributed to local emission sources, while in remote and rural areas, it can be referred to the impact of natural and anthropogenic sources (Tiwari et al. 2008; Keresztesi et al. 2019). Type and distribution of aerosol sources, transport, and scavenging processes of chemical species are just a few of the multiple factors that can influence the concentrations of chemical species in rainwater (Celle-Jeanton et al. 2009). Rainwater chemistry is also characterized by the complex interactions between the dynamics of clouds and microphysical processes, as well as different atmospheric chemical reactions that occur in-cloud and below-cloud (Herrera et al. 2009). The chemical composition of precipitation can also be affected by rainfall rate and amount, cloud base height, and the air mass back trajectories (Báez et al. 1997), varying from site to site and region to region (Pu et al. 2017), indicating the local influences and characteristics of atmospheric pollutants. Rainwater composition reflects the quality of the atmosphere during the occurrence of rainfall (Mahato et al. 2016).

The study of heavy metals concentration in rainwater has increased in the last decades due to their significant effects on the environment and human health (Báez et al. 2007). Heavy metals, such as Cd and Pb, are toxic and highly bioavailable, hence they can be easily accumulated by the biosphere and living organisms (Galloway et al. 1982). Anthropogenic activities, including mining, industry, coal burning, and automobile exhaust, are known to be the most significant sources of heavy metals concentration in precipitation. Trace metals are also deposited by precipitation to the surface of waters and soils and may have harmful effects on the ecosystem. Pollutants from the atmosphere are mainly deposited by the in-cloud scavenging (rainout) and below-cloud scavenging (washout) processes. According to Valenta et al. (1986) over 80% of wet deposited heavy metals are dissolved in rainwater, favoring the uptake of these pollutants by vegetation. Another factor influencing the rainwater chemistry and its composition are atmospheric circulations, by acting on radiation, temperature, and precipitation conditions (Falarz 2007). Foehn winds and the Foehn effect present in the studied area are of great importance, since the air masses are subjected to an additional warming and dehumidification, impacting the sea salt content in the rainwater. Emissions of air pollutants, especially the increased sulfur and nitrate concentrations in urban areas, are due to growth in population, industrialization, mining, energy consumption, transport, agricultural production, etc. Mining activities affect relatively small areas but can have a large local impact on the environment (Salomons 1995). Release of metals from mining sites occurs primarily through acid mine drainage and erosion of waste dumps and tailings deposits (Salomons 1995).

The role of local contribution versus long-range transport supply in the rainwater chemistry was investigated by the collection of rainwater samples during January 2014 and December 2017 at three sampling sites in the Southern and Western Carpathians. The studied area is one of the largest mining regions from Europe, known for its un-rehabilitated tailings ponds, tailings dams, and slag deposits, where thousands of tons of hazardous waste were gathered during mining operations. Surface mining activities are direct sources of air pollution and polluted atmospheric precipitation. While the European Union is trying to stop pollution and prevent the effects of global warming, the Romanian state is still confronting with serious problems regarding the closure and greening of the tailing ponds. One of the worst cases in the west of the country is the Boşneag decantation pond, Moldova Nouă, Caraş-Severin County. A mining operation started here 40 years ago, exploiting complex minerals, especially iron and copper (pyrite, chalcopyrite, etc.), while the banks of the Danube were nothing but settling ponds where thousands of tons of hazardous waste were gathered. Zinc, nitrates, and other metals have penetrated the soil and the water of the river. In the early 2000s, exploitation began to decline, and the exploitation was quickly shut down, leaving behind millions of tons of ore residues, which were used in the production process. The studied area represents a major pollution source, with thin particles of hazardous dust being carried in the atmosphere by the wind and transported to the neighboring shore in Serbia (Burlacu et al. 2017).

The main aim of this paper is the analysis of the effects of major ions and trace elements deriving from the tailing ponds, which have a high mineral charge, on the rainwater chemistry. Also, the study investigates the atmospheric circulation impact and the Foehn effect on the rainwater chemistry, taking into account the main air mass transport routes in order to explain the origin of the pollutants and relationship between major ions, providing a preliminary understanding of the influencing factors regarding the lack of sea salt contribution, and the presence of anthropogenic and crustal sources. The relationship between precipitation chemistry and pollutants deriving from anthropogenic activities was further examined using different atmospheric modeling techniques, in order to show the contribution of regional and continental air masses to the precipitation chemistry in one of the most polluted regions from Europe.

## Materials and methods

### Sampling sites and analysis

The studied area is situated in the Southern and Western Carpathians, South-West Romania, on the border with Serbia. This region is known for its great relief diversity, where all three classical forms of relief are present, but the mountains are predominant, representing 65.4% of the total surface of the studied region. The mountainous region has different characters due to its diverse geological and lithological structure, being represented by Banat Mountains, the western extremity of the Southern Carpathians (Țarcu, Godeanu, Cernei, and Mehedinți mountains) and the most southern part of the Western Carpathians (the southern peaks of Poiana Ruscă Mountains). The mountainous relief rises in altitude from west to east, culminating in the Godeanu Mountains, with their heights of 1600–2200 m, rising far above the southern part of the Poiana Ruscă Mountains (1355 m).

Due to its geographical position, not far from the Adriatic Sea and in the shelter of the Southern and Western Carpathians, the studied area’s climate is temperate continental, with sub-Mediterranean influences. This climate type is characterized by Atlantic air masses circulation and the invasion of Mediterranean air masses, giving a moderate character to the thermal regime, with frequent warm periods in the winter, early springtime, and relatively large amounts of rainfall. Precipitation amount increases from 700 mm in the low areas to 1400 mm in the Țarcu and Godeanu Mountains. The predominance throughout the year of the advent of wet air masses in the west and southwest, as well as the more intense frontal activity, gives the main feature of the climate.

**Fig. 1:**
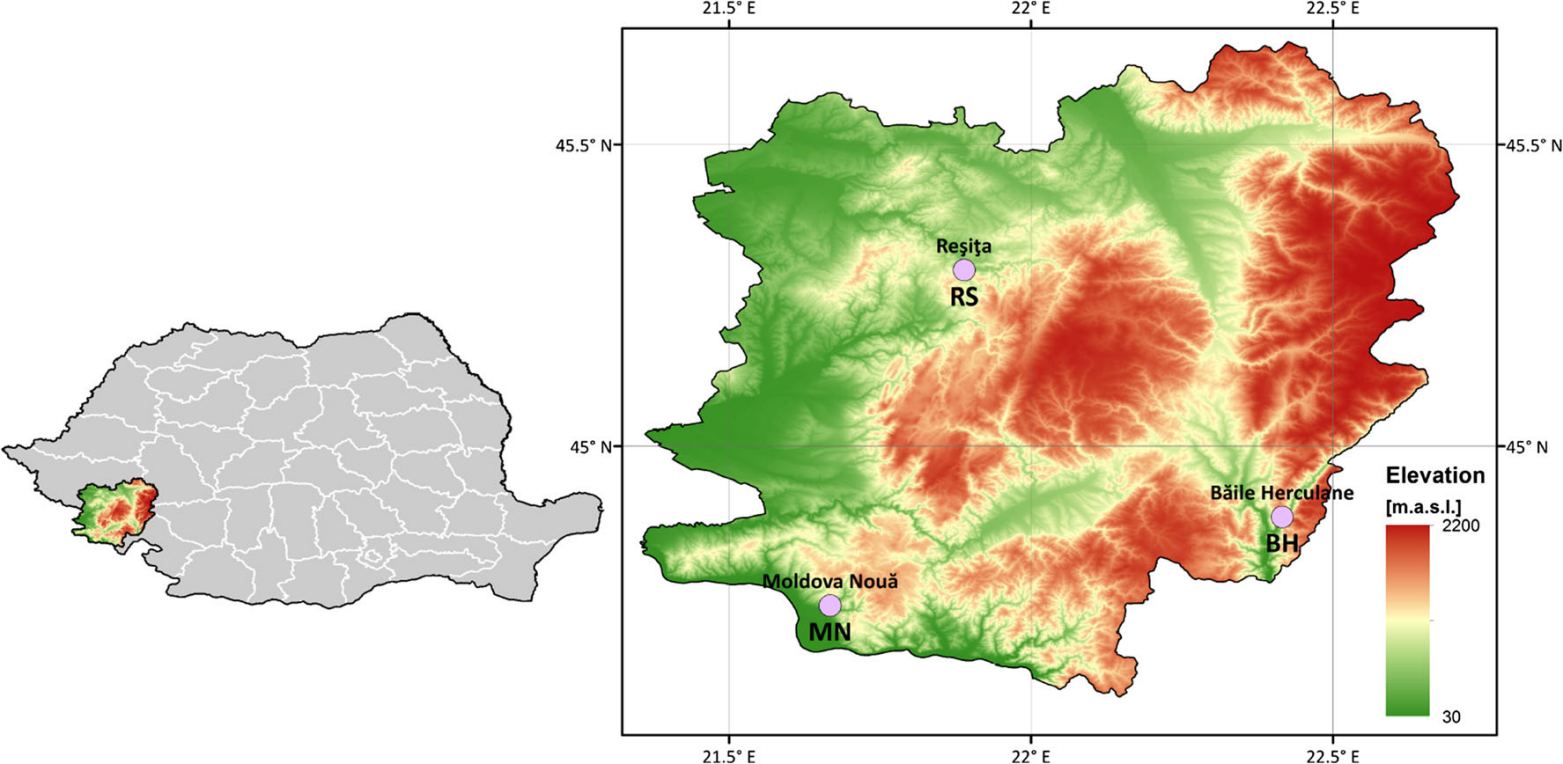
Sampling sites (RS, MN, BH), Southern and Western Carpathians, Romania

In order to analyze chemical composition of rainwater and the effects of the tailing ponds on the concentration of major ions and heavy metals, a total of 81 rainwater samples were collected at Reșița, 97 at Moldova Nouă and also 97 samples at Băile Herculane, from January 1, 2014 to December 31, 2017. The selected locations for rainwater sampling are significant in terms of mining and industry: Municipality of Reșița (RS) (45° 30′ N, 21° 88′ E) where steel and machinery industry is well developed, Moldova Nouă (MN) (44° 73′ N, 21° 66′ E) and Băile Herculane (BH) (44° 87′ N, 22° 41′ E). The geographical distribution of the sampling sites is shown in Fig. 1.

Rainwater samplers were placed about 1.5–2 m above ground, from the onset until the end of the precipitation event. In order to prevent/minimize any possible contamination, the samplers were thoroughly washed and rinsed before and after each collecting operation. After precipitation samples were collected, a small amount of thymol was added to all the samples to avoid biological degradation, and then samples were kept at 4 °C until ion composition analysis (SO_4_^2−^, NO_3_^−^, NO_2_^−^, Cl^−^, NH_4_^+^, Ca^2+^, Na^+^, K^+^, Mg^2+^). After each sampling, pH and conductivity were immediately measured, using digital pH meters standardized with 4.0 and 9.2 pH buffer solutions and conductivity meters. The anions (SO_4_^2−^, NO_3_^−^, NO_2_^−^) were analyzed by ion chromatograph (Dionex 2000i/SP) using a CO_3_^2−^/HCO_3_^−^ buffer as eluent (1.7 mM Na_2_CO_3_/1.8 mM NaHCO_3_), used in isocratic analysis, and 25 mM H_2_SO_4_ as regenerant. A total of 100 ppm stock solutions of sodium salts of each of the ions were prepared. The concentrations were calculated based on the peak area of the above-mentioned standards. After every five samples, peak response was checked, by running standards I triplicates. Recalibration was necessary if the deviation was more than 2%. Cations (Na^+^, Ca^2+^, Mg^2+^, and K^+^) and heavy metals (Pb, Ni, As, Cd) were measured by inductively coupled plasma-atomic emission spectroscopy (ICP-AES, iCAP 6300 Duo View ICP-AES Spectrometer) after acidification to pH < 2 with HNO_3_. The Cl^−^ and NH_4_^+^ were measured by U-VIS spectrometer method (Nicolet Evolution 100, 463 and 440 nm) (Nollet 2007; Szép et al. 2017).

### Data quality

To verify the completeness and correctness of precipitation data, ion balance technique was used. The data is generally considered acceptable, if the equivalent ratio of the sum of anions and sum of cations (Σ anions/Σ cations) is around one, with ion imbalances that does not exceed ± 25% (Keene et al. 1986; Wu et al. 2016). The ionic balance of total anions and cations for the precipitation samples was as follows: 0.75 ± 0.58 at RS, 0.77 ± 0.49 at MN and 0.77 ± 0.58 at BH. Eventual anion deficiencies observed in the ionic balances of the collected samples indicate the presence HCO_3_^−^, which can be estimated using the empirical method of Granat (1972), to lower the deviations from unity:

1 $$ \left[{\mathrm{HCO}}_3^{-}\right]={10}^{-11.24+\mathrm{pH}}\ \left(\mathrm{eq}/\mathrm{l}\right) $$

After taking under consideration the calculated HCO_3_^−^ concentrations, the ionic balance indicated significantly higher values at all 3 sampling sites being 0.82 ± 0.61 at RS, 0.90 ± 0.61 at MN, and 0.94 ± 0.70 at BH, respectively. Further anion deviation in ion balance might be due to the presence of organic compounds such as acetate, formate, and oxalate, emitted in the atmosphere by the biomass (Honório et al. 2010), that were not measured due to analytical limitations.

### Classification of atmospheric circulation types

**Fig. 2:**
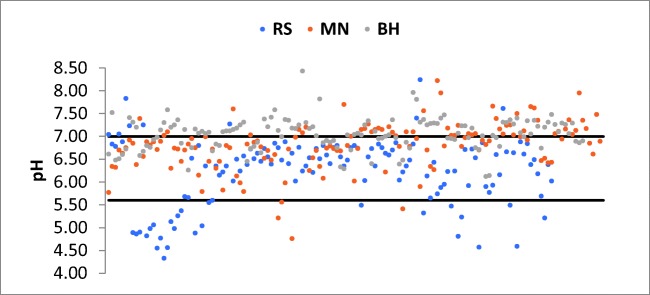
Distribution of rainwater pH at Resita (RS), Moldova Noua (MN), and Baile Herculane (BH)

Atmospheric circulations are important in the study of precipitation in certain areas as shown previously (Esteban et al. 2005; Haylock et al. 2008; Brisson et al. 2011; Fleig et al. 2015). For our purpose, we used a daily classification of circulation types (CT) in order to investigate the links between certain synoptic patterns and precipitation in southeastern Europe. In this manner, we used the mean sea-level pressure (MSLP) retrieved from ERA-Interim (Dee et al. 2011) as input and cost733class software (Philipp et al. 2016) in order to construct an objective and automatic classification of weather types. The classification chosen here is called GrossWetterTypen (GWT) which uses predefined thresholds in order to assign each day to a specific circulation type (Beck et al. 2007). A total of 27 weather types over the period 1979–2018 were obtained. We selected six weather types resembling to cyclonic formations upon the eastern Mediterranean basin that can lead to precipitation occurrence in the study area (Fig. 2). For each CT, we constructed the multi-annual mean of precipitation sums in order to visualize the distribution of rainfall upon southeastern Europe.

Daily gridded precipitation data from E-OBS (v20e) is used here at a spatial resolution of 0.1 degrees. This data is using the mean from 100-member ensemble in order to remove the uncertainties resulted from multiple interpolation methods (Cornes et al. 2018).

### Multivariate statistical analysis

Principal component analysis (PCA) is a multivariate statistical technique that can be applied in order to identify possible associations among the variables in a dataset, being frequently used to distinguish the relationships and the correspondence between different ionic species and elements in precipitation (Plaisance et al. 1996; Lara et al. 2001; Varmuza and Filzmoser 2010). A varimax rotation was applied to maximize the variance and to obtain a pattern of loadings for each factor (Cheng et al. 2011). The datasets were processed using the IBM SPSS version 23 statistical program.

### Cluster analysis

Meteorological classification refers to the identification of distinct patterns that influence climate-/weather-related variables (Riccio et al. 2007; Izquierdo et al. 2012). In order to classify the air mass origin arriving at a site, cluster analysis can be used.

However, cluster analysis does not provide further information on the geographical location of potential source regions (Izquierdo et al. 2012). In the present study, air mass movement is analyzed at the studied locations. During the simulations, the Hybrid Single Particle Lagrangian Integrated Trajectory Model (HYSPLIT) (Draxler and Rolph 2013) was used to quantitatively analyze the distribution of aerosols based on 1°×1° meteorological data (GDAS- Global Data Assimilation System) from NCEP. In order to find the dominant trajectories, cluster analysis was used (Anil et al. 2017).

## Results and discussions

### Major ion and heavy metal concentrations of rainwater

Statistical results of all analyzed ion and trace element concentrations in rainwater samples collected at three different sampling sites in the Southern Carpathians are given in Table 1, along with pH, volume-weighted mean (VWM), standard deviation (st. dev.), and standard error (st. error). Relatively high standard deviations of major ion concentrations at all sampling sites indicates great differences in the cation and anion concentrations over the precipitation events (Wu et al. 2012), while the lower VWM concentrations of ions than the arithmetic means suggest that lower rainfalls are usually accompanied by higher concentrations (Lü et al. 2017). This inverse relationship is probably due to the below-cloud removal process during precipitation events (De Souza et al. 2006; Wu et al. 2016).

**Table 1:** Volume-weighted mean (VWM) concentration of major ion (in μeq l^−1^), heavy metals (in μgl^−1^), and pH along with statistical results in rainwater samples

Component	pH	Na^+^	K^+^	Ca^2+^	Mg^2+^	SO_4_^2−^	Cl^−^	NO_2_^−^	NO_3_^−^	NH_4_^+^	HCO_3_^−^	H^+^	Pb	Ni	As	Cd
RS																
VWM	6.03	37.38	15.13	132.28	68.82	49.72	132.90	2.46	18.80	94.94	24.35	4.55	1.22	0.23	0.29	0.89
Average	6.18	38.21	15.77	158.48	78.10	61.87	129.39	2.60	20.65	99.23	31.39	3.17	1.28	0.20	0.34	0.74
St. dev.	0.75	40.94	22.96	135.00	73.50	48.61	55.20	3.30	18.87	60.19	98.63	7.02	0.77	0.24	0.21	0.60
St. error	0.07	3.69	2.07	12.17	6.63	4.38	4.98	0.30	1.70	5.43	8.89	0.63	0.07	0.02	0.02	0.05
Min.	4.33	3.44	0.97	5.29	1.65	10.41	20.03	0.11	0.18	0.33	0.12	0.01	0.17	0.02	0.06	0.16
Max.	8.24	247.94	129.42	799.94	473.98	291.48	400.53	22.21	116.06	262.22	1000.00	46.77	2.84	0.83	0.61	2.76
MN																
VWM	6.78	7.38	17.50	356.73	189.46	200.35	159.04	5.28	27.81	123.78	57.88	0.29	2.77	1.87	0.25	8.84
Average	6.81	35.87	21.20	367.31	219.42	240.91	160.72	5.39	27.73	130.00	68.16	0.45	2.84	1.10	0.26	8.68
St. dev.	0.52	54.76	21.13	252.54	234.56	155.92	74.77	6.29	32.00	94.46	106.50	1.58	1.80	2.64	0.18	3.03
St. error	0.04	4.63	1.79	21.34	19.82	13.18	6.32	0.53	2.70	7.98	9.00	0.13	0.16	0.24	0.02	0.27
Min.	4.76	3.52	1.79	28.44	3.79	20.82	20.03	0.07	0.35	16.63	0.33	0.01	0.74	0.05	0.00	1.99
Max.	8.22	430.54	135.40	1574.43	1026.95	874.43	600.80	36.10	212.89	398.04	954.99	17.38	7.29	8.91	0.54	15.32
BH																
VWM	7.02	14.17	17.46	382.16	201.86	204.87	162.65	7.25	12.80	93.13	86.44	0.13	4.15	1.40	0.38	7.73
Average	7.06	50.88	18.39	422.53	218.05	226.33	162.46	6.25	12.13	86.95	93.67	0.12	3.08	1.17	0.35	7.74
St. dev.	0.35	163.42	13.52	382.38	192.82	115.99	91.33	6.64	15.15	382.52	142.09	0.12	2.29	2.21	0.20	1.30
St. error	0.03	13.96	1.16	32.67	16.47	9.91	7.80	0.57	1.29	6.45	12.14	0.01	0.21	0.20	0.02	0.12
Min.	6.12	5.65	2.81	34.93	4.94	20.82	20.03	0.09	0.35	3.88	7.59	0.00	0.62	0.15	0.09	5.50
Max.	8.43	1360.17	72.89	2319.98	789.96	687.05	520.69	30.39	127.89	382.52	1548.82	0.76	8.90	7.46	0.56	10.35

Major ion concentration in the case of RS followed the descending sequence of Cl^−^ > Ca^2+^ > NH_4_^+^ > Mg^2+^ > SO_4_^2−^ > Na^+^ > HCO_3_^−^ > NO_3_^−^ > K^+^ > H^+^ > NO_2_^−^. The predominant cations were Ca^2+^, NH_4_^+^, and Mg^2+^ accounting for 83.84% of the total sum of cations, while Cl^−^ was the predominant anion and contributed 58.23% of the total mass of anions. Major ion concentration in rainwater collected at MN followed the descending sequence Ca^2+^ > SO_4_^2−^ > Mg^2+^ > Cl^−^ > NH_4_^+^ > HCO_3_^−^ > NO_3_^−^ > K^+^ > Na^+^ > NO_2_^−^ > H^+^, Ca^2+^ being the predominant cation accounting for 51.32% of the total mass of the cations, and SO_4_^2−^ being the most abundant anion, representing 44.49% of the total anionic mass. Almost the same can be said about the precipitation samples collected at BH, with the major ion concentration following the descending order: Ca^2+^ > SO_4_^2−^ > Mg^2+^ > Cl^−^ > NH_4_^+^ > HCO_3_^−^ > K^+^ > Na^+^ > NO_3_^−^ > NO_2_^−^ > H^+^. The dominant cations were Ca^2+^ and Mg^2+^ accounting for 53.91% and 28.47% of the total cationic mass, respectively. SO_4_^2−^ is the predominant anion, representing 43.22% of the total sum of anions. High concentrations of Ca^2+^ and Mg^2+^ collected at the sampling sites are due to the large contribution of soil dust from the un-rehabilitated tailing dams, from the dolomite limestones.

The dissolution of calcite (CaCO_3_), dolomite (MgCO_3_), and gypsum (CaSO_4_·2H_2_O) in the rainwater explains the high concentrations of Ca^2+^, Mg^2+^, and SO_4_^2−^ in the studied areas.

Among heavy metal content in rainwater, at RS, Pb presented the highest concentration, followed by Cd, while at MN and BH, Cd was the most abundant element, followed by Pb, the latter being 3, respectively 2 times lower than the concentrations measured for Cd. Ni and As were the least concentrated, having similar concentrations at RS, while at MN and BH Ni was 7 and 4 times higher than As, respectively.

### Variation of pH and acid neutralization

Naturally existing carbon dioxide can dissolve into the clouds and cloud droplets, resulting in the water carbonic acid forms. (Wang and Han 2011). At normal concentrations and pressures of carbon dioxide in the atmosphere, the pH of rain and snow usually tends to be 5.6 (Likens et al. 1979; Wang and Han 2011). pH values lower than 5.6 indicate a significant impact of anthropogenic activities on rainwater quality, while samples having pH values above 6 and 6.5 are suggesting a major quantity of alkaline species in the atmosphere and rainwater of the studied area (Cao et al. 2009). The pH values of individual precipitation events between 2014 and 2017 varied from 4.33 to 8.43 for the entire studied region with an average of 6.70. About 7.32% of the rainwater events had pH values less than 5.6, 56.83% of the total samples had pH values between 5.6 and 7, while 35.85% presented higher values than 7, with 0.73% of the total samples ranging between 7 and 8.43. The distribution of rainwater pH is showed in Fig. 2. Individual pH values at the sampling sites were also analyzed (Table 1). The most acidic pH (values < 5.6) were found at RS, representing 20.16% of the collected samples. In the case of MN, 2.80% of the total samples yielded pH values lower than 5.6, while at BH station, none of the rain events had pH values lower than 5.6.

At RS and MN, the majority of the precipitation events had pH values between 5.6 and 7, representing 72.87% and 59.44%, respectively. BH had more alkaline pH, with 60.87% of the total samples ranging between 7.00 and 8.43. High pH values can result from the dissolution of windblown dust and can be derived from the weathering of carbonate with a high CaCO_3_ content (Wu et al. 2012).

The relatively high mean pH values measured in the studied region are mainly caused by the neutralization of acidity in rainwater, instead of lack of acidic compounds (Al-Momani et al. 2000).

This relationship is further confirmed by the neutralization percentage of alkaline species (Ca^2+^, Mg^2+^, and NH_4_^+^), which is estimated using the neutralization factors (NF) (Kulshrestha et al. 1995; Zhang et al. 2007):

2 $$ {\mathrm{NF}}_{\mathrm{xi}}=\frac{\left[{X}_{\mathrm{i}}\right]}{\left[{\mathrm{SO}}_4^{2-}\right]+\left[{\mathrm{NO}}_3^{-}\right]} $$

where [*X*_i_] is the concentration of the alkaline component (Ca^2+^, NH_4_^+^, Na^+^, K^+^, Mg^2+^) expressed in μeq/L.

As presented in Table 2, Ca^2+^ is the most dominant neutralizing compound in precipitation at the three sampling sites, the average NF_Ca_ values ranging between 1.56 at MN and 2.16 at CS for the 2014–2017 period. Mg^2+^ and NH_4_^+^ were also significant neutralizing agents, their average NF values varied from 0.52 to 1.51. Neutralization effect of K^+^ and Na^+^ could be negligible, their NF values ranging between 0.09 and 0.61.

**Table 2:** Neutralization factors of the major ions and AP/NP ratio in rainwater samples

	NF_NH4_	NF_Ca_	NF_Na_	NF_K_	NF_Mg_	AP/NP
RS						
Mean value	1.51	2.16	0.61	0.23	1.01	0.31
St. dev.	1.12	1.62	0.75	0.29	0.94	0.31
Range	0.01–5.74	0.04–8.67	0.03–4.45	0.01–1.27	0.03–4.15	0.06–1.92
MN						
Mean value	0.60	1.56	0.13	0.10	0.77	0.54
St. dev.	0.50	1.17	0.13	0.10	0.81	0.39
Range	0.03–2.39	0.21–5.57	0.02–0.81	0.01–0.54	0.03–3.08	0.14–1.65
BH						
Mean value	0.52	1.73	0.14	0.09	0.99	0.61
St. dev.	0.62	1.58	0.12	0.07	1.33	0.57
Range	0.02–3.35	0.18–9.60	0.02–0.61	0.01–0.29	0.03–9.20	0.05–2.27

According to Kaya and Tuncel (1997) and Wu et al. (2016), the acidification potential depends on the concentration of H_2_SO_4_, HNO_3_, and other organic acids in rainwater, while the neutralization potential on the competence of alkaline constituents to neutralize the acidic species (Rastogi and Sarin 2005). The acidic potential is the sum of the concentrations of NO_3_^−^ and nss-SO_4_^2−^ and neutralization potential is the sum of the concentrations of NH_4_^+^, nss-Ca^2+^, nss-Mg^2+^, and nss-K^+^ (Kumar et al. 2002). Acidic potential can be used as a tracer of anthropogenic activities, while the neutralization potential as an indicator of air mass transport (Fujita et al. 2000). The AP/NP ratio was estimated for all three sampling locations and results are shown in Table 2. At all sampling sites, the average NP value is higher than the AP value, the ratio of AP/NP varying from 0.31 at RS to 0.61 at BH.

To further asses the relative contribution of different acidic or neutralizing compounds in rainwater, the H^+^/(NO_3_^−^ + SO_4_^2−^), (NO_3_^−^ + Cl^−^)/(SO_4_^2−^), (Ca^2+^ + Mg^2+^ + NH_4_^+^)/(NO_3_^−^ + SO_4_^2−^), SO_4_^2−^/ NO_3_^−^, NH_4_^+^/NO_3_^−^, and NH_4_^+^/SO_4_^2−^ ratios were calculated (Table 3) (Tiwari et al. 2016).

**Table 3:** Ionic ratios among measured ions

	$\frac{{\mathrm{H}}^{+}}{{\mathrm{NO}}_3^{-}+{\mathrm{SO}}_4^{2-}}$	$\frac{{\mathrm{SO}}_4^{2-}}{{\mathrm{NO}}_3^{-}}$	$\frac{{\mathrm{NH}}_4^{+}}{{\mathrm{NO}}_3^{-}}$	$\frac{{\mathrm{NH}}_4^{+}}{{\mathrm{SO}}_4^{2-}}$	$\frac{{\mathrm{Ca}}^{2+}+{\mathrm{Mg}}^{2+}+{\mathrm{NH}}_4^{+}}{{\mathrm{NO}}_3^{-}+{\mathrm{SO}}_4^{2-}}$	$\frac{{\mathrm{NO}}_3^{-}+{\mathrm{Cl}}^{-}}{{\mathrm{SO}}_4^{2-}}$	$\frac{{\mathrm{NO}}_3^{-}+{\mathrm{SO}}_4^{2-}}{{\mathrm{Ca}}^{2+}+{\mathrm{Mg}}^{2+}}$
RS	0.08	26.47	36.30	2.35	4.68	3.72	0.79
MN	0.002	35.94	15.61	0.68	2.93	1.02	0.90
BH	0.001	68.31	22.44	0.55	3.23	0.98	1.00

The ratio H^+^/(NO_3_^−^ + SO_4_^2−^), known as fractional acidity (FA), indicates whether or not the rainwaters acidity is neutralized (Balasubramanian et al. 2001; Anatolaki and Tsitouridou 2009). If the value of FA is close to one, the acidity in precipitation caused by strong acids (H_2_SO_4_; HNO_3_) is not neutralized at all. The average mean FA ratio varied from 0.001 to 0.08, being far from unity, which indicates that on average, almost 99% of the inorganic acidity in precipitation is neutralized. The ratio of (NO_3_^−^ + Cl^−^)/(SO_4_^2−^) ranged between 0.98 (BH) and 3.72 (RS) in the studied area, indicating that where higher ratios were found (RS, MN), nitric and hydrochloric acids have a larger contribution, while at BH where this ratio presented lower values, the influence of sulfuric acid is more significant (Singh et al. 2007). Another indicator for acidity is the ratio of (SO_4_^2−^ + NO_3_^−^)/(Ca^2+^ + Mg^2+^), assuming that rainwaters acidity is primarily due to sulfate and nitrate (Jawad Al Obaidy and Joshi 2006). A ratio lower than one indicates the alkaline nature of precipitation, while a value greater than unity the presence of free anions (Jawad Al Obaidy and Joshi 2006). The values of the above-mentioned ratio presented values between 0.79 (RS) and 1.00 (BH), precipitation at RS and MN, being more alkaline than at BH, where the acidic components in rainwater are more significant. According to Tiwari et al. (2012), the equivalent ratio of (Ca^2+^ + Mg^2+^ + NH_4_^+^)/(NO_3_^−^ + SO_4_^2−^) can be used as an indicator in the evaluation of anthropogenic activity contribution to the acidity of rainwater. This ratio at all sampling sites had high values, varying from 2.93 (MN) to 4.68 (RS), indicating that Ca^2+^, Mg^2+^, and NH_4_^+^ had a significant role in the neutralization process, in the form of CaSO_4_, (NH_4_)_2_SO4, and MgSO_4_. The ratio of NH_4_^+^/SO_4_^2−^ and NH_4_^+^/NO_3_^−^ is a possible indicator of NH_4_NO_3_ and (NH_4_)_2_SO_4_ (Seinfeld 1986). Elevated values of the SO_4_^2−^/NO_3_^−^ ratio showed an excess of sulfate over nitrate, values ranging between 26.47 and 68.31, indicating anthropogenic sources in the atmospheric precipitation of industrial areas (Migliavacca et al. 2005). Use of fertilizers containing (NH_4_)_2_SO_4_ and NH_4_NO_3_ can be converted into NH_3_, which in the atmosphere can act as a neutralizing agent (Al-Momani et al. 1995a; Kaya and Tuncel 1997; Seinfield and Pandis 1998; Migliavacca et al. 2005).

### Wet deposition rates of major ions and heavy metals

Wet deposition is an effective route of pollutants removal from the atmosphere, while quantitative measurement of wet deposition helps to identify the relative contributions of the various natural and anthropogenic sources (Tiwari et al. 2016). The annual wet deposition (WD) flux is expressed in kg ha^−1^ year^−1^, and is calculated taking under consideration the VWM (mg/L) and the annual rainfall (RF) amount, using the following equation:

3 $$ \mathrm{WD}\left({\mathrm{kgha}}^{-1}\ {\mathrm{yr}}^{-1}\right)=\mathrm{VWM}\left(\mathrm{mg}\ {\mathrm{L}}^{-1}\right)\times \frac{\mathrm{RF}}{100} $$

**Fig. 3:**
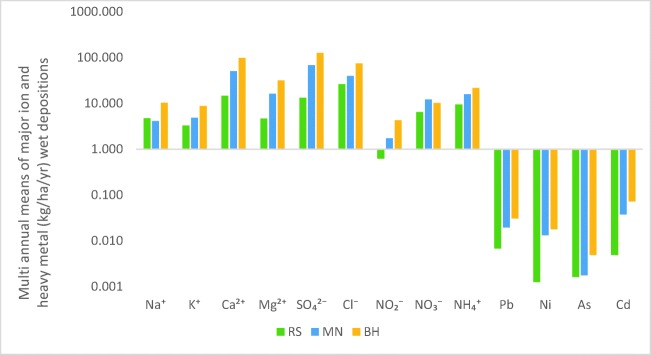
The average multi annual wet flux depositions (kg ha^–1^ year^–1^) of major ions at the studied sampling sites

The multi-annual WD in precipitation over the 4-year period (2014–2017) at the sampling sites of RS, MN, and BH is displayed in Fig. 3 and varied from site to site due to different precipitation amounts and different influences of major ion and heavy metal sources.

In the case of heavy metals, Cd had the highest wet deposition rates, 0.04 kg ha^−1^ year^−1^ at MN and 0.07 kg ha^−1^ year^−1^ at BH. In the case of Pb, the most elevated values were also observed at MN and BH, presenting 0.02 kg ha^−1^ year^−1^ and 0.03 kg ha^−1^ year^−1^, respectively. Ni and As had insignificant wet deposition fluxes. WD fluxes for acidic species, such as SO_4_^2−^ and Cl^−^, were the highest when the precipitation amount increased, while in the case of lower precipitation amounts, alkaline species had lower WD rates (Tiwari et al. 2016). The highest multi-annual mean WD flux for both anions and cations was measured at BH, deposition rates here being with ~ 33% and 147% greater than at MN and RS, respectively. The highest WD flux for SO_4_^2−^ (125.86 kg ha^−1^ year^−1^) and Cl^−^ (73.75 kg ha^−1^ year^−1^) was measured at BH, followed by MN with 67.74 kg ha^−1^ year^−1^ for SO_4_^2−^ and 39.69 kg ha^−1^ year^−1^ for Cl^−^. In the case of RS, Cl^−^ had the highest deposition flux, accounting for 26.01 kg ha^−1^ year^−1^. Regarding the cations, at all sampling sites, Ca^2+^ had the highest wet deposition values, being 97.95 kg ha^−1^ year^−1^, 50.33 kg ha^−1^ year^−1^, and 14.63 kg ha^−1^ year^−1^ at BH, MN, and RS, respectively.

The results regarding the WD rates of major ions correlate with the results obtained for NFs, explaining the greater concentration of acidic species at BH and MN, which may be due to coal combustion and the use of chloride in the metallurgic industry. Another cause of higher wet deposition rate of sulfate is due to the size of this ion in the atmosphere (Al-Momani et al. 1995a). Being a secondary particle, this ion is produced by the gas to particle conversion process, leading to the formation of ultrafine particles, which can grow via coagulation (Al-Momani et al. 1995b). Sulfate is removed most efficiently by wet deposition, through the in-cloud scavenging mechanism, causing higher WD rates.

### Assessment of ecological risks and potential effects of increased heavy metal concentration

Trace metals occur in many environmental matrices, and can be naturally found in the Earth’s crust (Chubaka et al. 2018). According to the World Health Organization (WHO) Pb, Cd, As, and Ni are classified as highly toxic to humans. Concentrations of these metals can also be found in rainwater, and are emitted to the atmosphere from metal smelters and waste processing plants, but mining and industrial activities also play a significant role in increasing the concentrations of these metals in the environment (Morais et al. 2012; Malassa et al. 2014; Chubaka et al. 2018). High concentrations of heavy metals deposited into common sources of drinking water, such as lakes and streams. can increase the concentrations of these metals above the acknowledged safety limits, affecting the ecosystem, humans or other organisms (Galloway et al. 1982). The toxicity potential (TP) of rainwater chemistry and of heavy metals in wet deposition can be calculated using the recommended upper limits for metal concentrations to observed median values in wet deposition (Galloway et al. 1982):

4 $$ \mathrm{TP}=\frac{\mathrm{Concentration}\ \mathrm{of}\ \mathrm{metal}\ \mathrm{in}\ \mathrm{wet}\ \mathrm{deposition}}{\mathrm{Recommended}\ \mathrm{upper}\ \mathrm{limits}\ \mathrm{for}\ \mathrm{metal}\ \mathrm{concentration}} $$

TP values > 1 imply potential toxic deposition. In order to define the toxicity potential of wet deposited heavy metals from rainwater, the maximum contaminant level (MCL) value was used. According to the Drinking Water Standards and Health Advisories (USEPA 2012), MCL represents the highest level of a contaminant that is allowed in drinking water. This value is 0.015 mg/L, 0.005 mg/L, and 0.01 mg/L for Pb, Cd, and As, respectively. Ni does not have a MCL values, but according to Chubaka et al. (2018), the health limit for Ni is 0.02 mg/L. Results have shown that Cd had the highest values at all sampling sites (Table 4), at MN and BH, the TP being 7 and 14 times higher than the reference value, respectively. Pb also exhibited higher values than 1 at MN and BH, suggesting a potential toxicity, as for As and Ni, values were much lower. TP values > 1 in the case of MN and BH are suggesting an elevated risk for human health in case of rainwater ingestion, especially in rural areas, where harvested rainwater is still used in households, but in order to establish if heavy metals concentration found in rainwater is affecting human health additional research is mandatory.

**Table 4:** Toxicity potential for heavy metals in wet deposition

	Pb	Ni	As	Cd
RS	0.45	0.06	0.16	0.98
MN	1.3	0.66	0.18	7.49
BH	2.04	0.89	0.48	14.33

In order to further evaluate the contamination of precipitation, the contamination factor and the contamination degree were calculated. Results are shown in Table 5. The contamination factor represents the ratio between the concentration of heavy metal measured in rainwater divided by the background value for the respective metal (Liu et al. 2005):

**Table 5:** Contamination factors and contamination degree of heavy metal concentrations in rainwater

	Contamination factor (${C}_f^i\Big)$				Contamination degree (*C*_d_)
	Pb	Ni	As	Cd	
RS	2.53	−5.86	2.57	5.16	4.39
MN	2.74	−0.72	3.68	1.54	7.24
BH	3.90	−1.13	2.22	1.20	6.19

5 $$ {C}_{\mathrm{f}}^{\mathrm{i}}=\frac{C_{\mathrm{i}}}{C_{\mathrm{b}}} $$

where *C*_i_ is the concentration of an individual metal measured in rainwater, while *C*_b_ represents the background concentration calculated for the individual metal. The background value of each metal was determined using the relationship between statistical estimators (mean ± 2σ), after extreme values were removed from the dataset, in order to assure the correctness and the applicability of this method (Reimann et al. 2005; Hernández-Crespo and Martín 2015).

The contamination degree can be calculated by summing all contamination factors for all metals (Liu et al. 2005):

6 $$ {C}_{\mathrm{d}}=\sum \limits_{\mathrm{i}=1}^{\mathrm{n}}{C}_{\mathrm{f}}^{\mathrm{i}} $$

The method described above was also used by Hakanson (1980) and Luo et al. (2007) to calculate the soil contamination. This work uses the same method, but it was adapted to assess the contamination with heavy metals in rainwater. According to Hakanson (1980), four categories of ${C}_{\mathrm{f}}^{\mathrm{i}}$ and four categories of *C*_d_ can be defined. Regarding contamination factors, a value < 1 indicates low contamination, values between 1 and 3 are known as moderate, 3 ≤ ${C}_{\mathrm{f}}^{\mathrm{i}}$ < 6 indicates considerable, while values > 6 are a sign of a very high contamination. The classes defined for *C*_d_ are the same, but having different intervals: *C*_d_ < 5–low contamination, 5 ≤ *C*_d_ < 10–moderate contamination, 10 ≤ *C*_d_ < 20 considerable contamination, while with values > 20 represent a high level of contamination (Hakanson 1980).

Based on the individual contamination factor of heavy metal concentration, rainwater collected at RS was moderately contaminated with Pb and considerably contaminated with Cd. Precipitation samples collected at MN were found to be moderately contaminated with Pb and Cd, while contamination with As is considerable. Contamination factors for BH showed that rainwater is slightly contaminated with As and Cd, while the ${C}_{\mathrm{f}}^{\mathrm{i}}$for Pb posed as considerable. The overall precipitation contamination with heavy metals at the three sampling sites was assessed by the degree of contamination, which showed that precipitation at MN and BH is considerably contaminated, while at RS, the *C*_d_ lies within the class of moderate contamination.

### Origins of major ions in rainwater

#### Marine and non-marine influence and their relationship with atmospheric circulations

In order to assess the main sources of chemical compositions in rainwater, including sea salts, terrestrial dust from wind erosion, mining, biogenic material, and various anthropogenic activities, enrichment factors (EFs), sea salt fractions (SSF), and non-sea salt fractions (NSSF) were calculated. Enrichment factors are usually used to investigate the potential sources of elements in rainwater (Okay et al. 2002; Cao et al. 2009; Wu et al. 2016). Since Na^+^ is known to be the best tracer for seawater, and assuming all Na^+^ is of marine origin, it was used as reference element. In order to calculate the EF values and to quantify the sea salt (SSF) and non-sea salt (NSSF) contributions to rainwater, the following equations were used (Keene et al. 1986; Kulshrestha et al. 1996; Conradie et al. 2016):

7 $$ \mathrm{Enrichemen}\ \mathrm{Factor}\ (X)=\frac{{\left(\raisebox{1ex}{$X$}\!\left/ \!\raisebox{-1ex}{$\mathrm{Na}$}\right.\right)}_{\mathrm{rain}}}{{\left(\raisebox{1ex}{$X$}\!\left/ \!\raisebox{-1ex}{$\mathrm{Na}$}\right.\right)}_{\mathrm{sea}}} $$

8 $$ \%\mathrm{SSF}=\frac{100\times {\left(\mathrm{Na}\right)}_{\mathrm{rain}}\times {\left(\raisebox{1ex}{$X$}\!\left/ \!\raisebox{-1ex}{$\mathrm{Na}$}\right.\right)}_{\mathrm{sea}}}{(X)_{rain}} $$

9 $$ \% NSSF=100-\mathrm{SSF} $$

where *X* is the concentration of the respective ion. The results are shown in Table 6. The enrichment factor is often used to differentiate aerosol sources, if the EF value is much smaller than 1 or much greater than 1 is considered concentrated or diluted relative to the reference source (Okay et al. 2002; Wu et al. 2016). EF values for K^+^, Ca^2+^, Mg^2+^, and SO_4_^2−^ were significantly higher than one, reflecting strong terrestrial influence, while although EF values for Cl^−^ were higher than 1, but still much lower than the EF values for the other ions, it can indicate a small contribution from seawater (Wu et al. 2016).

**Table 6:** Equivalent ratios of different components with reference to Na^+^ in rainwater

	$\frac{{\mathrm{Cl}}^{-}}{{\mathrm{Na}}^{+}}$	$\frac{{\mathrm{K}}^{+}}{{\mathrm{Na}}^{+}}$	$\frac{{\mathrm{Ca}}^{2+}}{{\mathrm{Na}}^{+}}$	$\frac{{\mathrm{Mg}}^{2+}}{{\mathrm{Na}}^{+}}$	$\frac{{\mathrm{SO}}_4^{2-}}{{\mathrm{Na}}^{+}}$
Seawater	1.16	0.02	0.04	0.22	0.12
RS					
Mean value (rainwater)	6.35	0.50	6.30	3.05	2.55
%SSF	34.91	5.20	1.08	12.26	7.81
%NSSF	65.09	94.80	98.92	87.74	92.19
%EF	5.48	22.87	143.27	13.45	20.38
MN					
Mean value (rainwater)	8.06	0.93	17.53	9.43	10.88
%SSF	26.77	3.57	0.43	3.94	1.88
%NSSF	73.23	96.43	99.57	96.06	98.12
%EF	6.95	42.12	398.44	41.56	87.02
BH					
Mean value (rainwater)	7.91	0.91	18.60	9.47	12.02
%SSF	20.23	3.28	0.31	3.20	1.44
%NSSF	79.77	96.72	99.69	96.80	98.56
%EF	6.82	41.29	422.75	41.72	96.13

The average values of ionic ratios of Cl^−^, K^+^, Ca^2+^, Mg^2+^, and SO_4_^2−^ with respect to Na^+^ in rainwater were calculated and compared with the respective ratios in seawater. The much higher ionic ratio values found for rainwater suggest the strong contribution of other sources, such as local natural emissions, soil dust, and anthropogenic activities (Samara and Tsitouridou 2000). The results for SSFs and NSSFs at all sampling sites show that all Cl^−^, K^+^, Ca^2+^, Mg^2+^, and SO_4_^2−^ mainly have non-marine origins. In the case of Cl^−^, the highest SSF was estimated at RS, ~ 35%. The chloride non-sea salt fraction could be attributed to various anthropogenic activities, such as automobile exhaust and iron and steel production (Xu et al. 2009; Wu et al. 2016).

Dissolution of evaporite minerals, such as halite and sylvite, from soil dust can be attributed as natural sources to NSSF Cl^−^ (Xu et al. 2009). SO_4_^2−^ showed the high dominance of non-marine source, with a NSSF value of 92.19%, 98.12%, and 98.56% at RS, MN, and BH, respectively. These values are representing anthropogenic origin, related to the considerable energy consumption, coal combustion, and high industrialization rate in the studied areas. K^+^, Ca^2+^, and Mg^2+^ are all originated from terrestrial sources, resulting from soil dust from the un-rehabilitated tailing dams and from the dissolution of dolomite limestones, fertilizers, and agricultural activities.

These results are also sustained by the weak correlations between Na^+^ and Cl^−^ indicating fractionation of sea salt and modification by non-marine constituents (Tables 7, 8, and 9), as the sampling sites are approximately 540 km away from the Adriatic Sea and 1240 km away from the Mediterranean Sea. The Foehn effect also has a significant contribution to the presence of weak correlations between sodium and chloride.

**Table 7:** Spearman correlation coefficients for ionic constituents and trace elements in precipitation

	Na^+^	K^+^	Ca^2+^	Mg^2+^	NH_4_^+^	SO_4_^2−^	Cl^−^	NO_2_^−^	NO_3_^−^	HCO_3_^−^	Pb	Ni	As	Cd	Cond.
RS															
Na^+^	1														
K^+^	*0.53*	1													
Ca^2+^	0.28	*0.43*	1												
Mg^2+^	0.14	0.32	*0.77*	1											
NH_4_^+^	0.11	*0.41*	*0.39*	0.33	1										
SO_4_^2−^	*0.37*	*0.39*	*0.47*	0.34	*0.35*	1									
Cl^−^	0.13	0.10	0.34	0.32	0.12	0.31	1								
NO_2_^−^	0.27	0.25	0.24	0.15	0.27	*0.35*	0.19	1							
NO_3_^−^	**−** *0.35*	0.07	0.07	0.26	0.26	0.03	0.13	− 0.10	1						
HCO_3_^−^	− 0.11	0.04	0.12	0.10	0.24	0.14	0.11	0.29	*0.38*	1					
Pb	0.14	0.26	0.16	*0.66*	*0.35*	*0.58*	*0.53*	*− 0.38*	*0.40*	− 0.23	1				
Ni	*0.56*	*0.46*	− 0.19	0.28	*0.45*	*0.63*	*0.50*	0.32	0.25	− 0.12	− 0.25	1			
As	0.27	0.03	*0.52*	0.20	0.30	− 0.05	− 0.32	− 0.01	0.19	0.20	0.20	− 0.22	1		
Cd	0.20	0.24	−0.33	0.02	0.18	*0.49*	*0.58*	0.27	*0.66*	− 0.27	0.16	*0.38*	*0.60*	1	
Cond.	*0.36*	*0.62*	*0.57*	*0.48*	*0.49*	*0.68*	0.29	*0.37*	0.18	*0.35*	*0.42*	0.07	*− 0.40*	0.19	1

MN															
Na^+^	1														
K^+^	*0.56*	1													
Ca^2+^	0.30	0.17	1												
Mg^2+^	0.32	0.14	*0.78*	1											
NH_4_^+^	0.04	0.21	0.32	0.31	1										
SO_4_^2−^	*0.58*	*0.35*	*0.42*	*0.40*	0.22	1									
Cl^−^	*0.42*	0.26	*0.49*	*0.43*	*0.36*	0.24	1								
NO_2_^−^	0.12	0.16	0.21	0.20	*0.71*	0.27	0.04	1							
NO_3_^−^	− 0.09	0.05	0.29	0.27	− 0.16	− 0.04	− 0.06	*− 0.39*	1						
HCO_3_^−^	0.20	0.21	− 0.08	− 0.13	0.25	0.26	0.01	0.22	− 0.24	1					
Pb	*0.59*	*0.47*	*0.49*	*0.65*	0.33	*0.53*	*0.39*	0.14	*0.47*	*0.47*	1				
Ni	*0.73*	*0.54*	*0.63*	*0.64*	0.29	*0.78*	*0.41*	− 0.21	*0.51*	0.10	0.17	1			
As	0.20	0.02	*0.57*	*0.36*	0.08	*0.50*	*0.44*	0.11	*− 0.50*	*0.61*	*0.46*	0.02	1		
Cd	*0.78*	0.27	*0.51*	*0.89*	*0.53*	*0.73*	0.28	0.34	*− 0.45*	0.12	*0.62*	0.27	*0.40*	1	
Cond.	*0.45*	*0.37*	*0.45*	*0.46*	0.22	*0.87*	*0.37*	0.15	0.14	0.22	*0.45*	*0.58*	*0.53*	*0.67*	1
BH															
Na^+^	1														
K^+^	0.19	1													
Ca^2+^	0.25	*− 0.45*	1												
Mg^2+^	0.20	*− 0.43*	*0.80*	1											
NH_4_^+^	− 0.31	− 0.16	− 0.10	0.09	1										
SO_4_^2−^	0.01	0.08	*0.40*	0.11	− 0.33	1									
Cl^−^	0.18	−`0.30	0.24	*0.44*	0.09	− 0.11	1								
NO_2_^−^	− 0.23	− 0.09	− 0.01	0.01	0.19	0.29	− 0.07	1							
NO_3_^−^	− 0.21	0.01	0.08	0.22	0.09	0.21	0.22	− 0.01	1						
HCO_3_^−^	0.00	0.30	0.21	− 0.11	0.20	0.15	0.17	0.12	0.19	1					
Pb	0.23	*0.38*	*0.37*	*0.49*	− 0.29	*0.77*	0.12	0.07	0.09	*− 0.37*	1				
Ni	0.17	0.33	0.27	0.32	*0.45*	*0.78*	*0.40*	0.07	*− 0.43*	*− 0.41*	*0.54*	1			
As	0.32	0.11	0.54	0.04	0.14	− 0.07	*0.59*	*0.50*	− 0.29	− 0.04	0.04	0.32	1		
Cd	*0.41*	*0.35*	*0.77*	*0.35*	0.01	*0.65*	0.21	0.25	*− 0.52*	0.16	0.19	0.19	*0.90*	1	
Cond.	0.05	0.19	*0.85*	0.16	0.25	*0.77*	0.13	0.21	0.27	0.34	*0.56*	*0.44*	− 0.31	*0.54*	1

Values with R > 0.35 were considered significant at 99% confidence level

**Table 8:** Varimax rotated factor loadings, total variance, and ionic sources at the studied sampling sites

RS	F1	F2	F3	F4
Na^+^	.041	.274	.384	−.681
K^+^	.195	− .128	*.899*	−. 150
Ca^2+^	*.865*	.291	.116	− .127
Mg^2+^	*.916*	.096	.063	.173
SO_4_^2−^	.224	*.729*	.212	− .336
Cl^−^	.261	*.730*	− .099	.274
NO_3_^−^	.088	.190	.135	*.798*
NH_4_^+^	− .047	.494	*.694*	.157
% Total variance	21.94	19.11	19.07	17.26
Possible source	Crustal	Anthropogenic	Agricultural	Traffic
MN	F1	F2	F3	F4
Na^+^	.134	.515	*.575*	.374
K^+^	.063	.007	.048	*.976*
Ca^2+^	*.758*	− .038	.327	.069
Mg^2+^	*.896*	− .084	.006	.083
SO_4_^2−^	*.699*	.188	.144	− .004
Cl^−^	.219	− .203	*.881*	− .013
NO_3_^−^	.363	*.736*	.014	.021
NH_4_^+^	.321	*− .752*	.199	.034
% Total variance	27.15	18.23	15.97	13.82
Possible source	Mixed/crustal	Anthropogenic	Marine	Agricultural
BH	F1	F2	F3	F4
Na^+^	− .132	.359	*.767*	− .227
K^+^	− .447	*.662*	.214	.067
Ca^2+^	*.829*	− .123	.223	.075
Mg^2+^	*.803*	− .205	.079	.082
SO_4_^2−^	*.762*	.433	− .159	− .135
Cl^−^	.317	− .182	*.795*	.177
NO_3_^−^	.063	*.832*	− .018	.036
NH_4_^+^	.030	.066	− .016	*.973*
% Total variance	27.95	19.28	16.86	12.33
Possible source	Mixed/crustal	Anthropogenic	Marine	Agricultural

The italicized values represent the segnificant factor loadings

**Table 9:** Varimax-rotated factor loadings, total variance and possible sources of heavy metals in RS, MN and BH rainwater

RS	F1	F2	F3
Pb	.033	.155	*.987*
Ni	*.877*	− .432	− .052
As	.005	*.969*	.178
Cd	*.895*	.383	.108
% Total variance	39.28	32.42	25.53
Possible source	Coal combustion	Smelters	Non-ferrous metal production/combustion
MN	F1	F2	F3
Pb	.171	.049	*.983*
Ni	*.939*	.035	.231
As	.176	*.980*	.049
Cd	*.915*	.297	.062
% Total variance	44.51	26.33	25.65
Possible source	Coal combustion	Smelters	Non-ferrous metal production/combustion
BH	F1	F2	F3
Pb	.120	.167	*.978*
Ni	.234	*.954*	.165
As	*.758*	.561	.263
Cd	*.977*	− .155	− .064
% Total variance	39.94	31.89	26.44
Possible source	Smelters	Oil-fired furnaces	Non-ferrous metal production/combustion

The italicized values represent the segnificant factor loadings

**Fig. 4:**
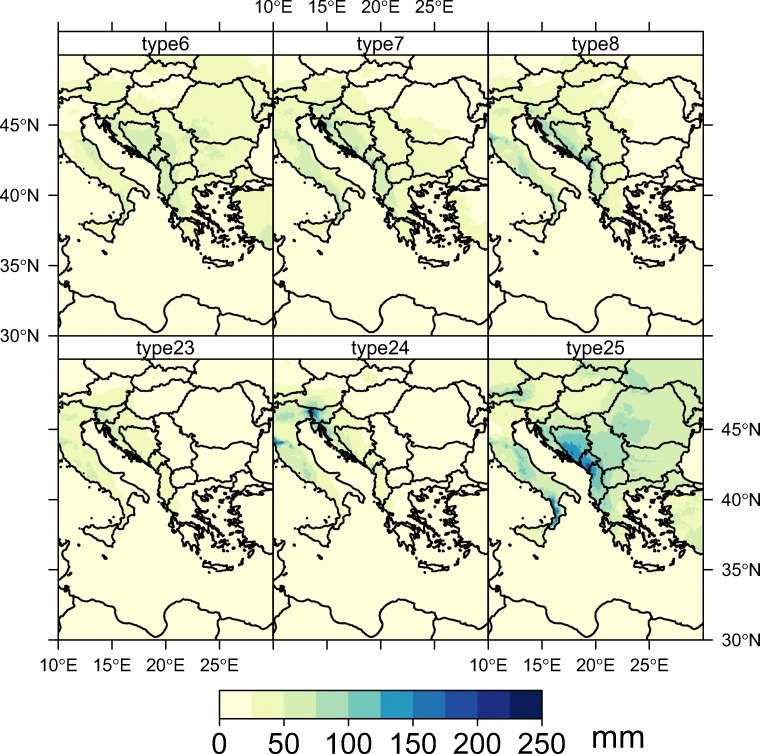
Multi-annual means of precipitation sums according to each cyclonic weather type

**Fig. 5:**
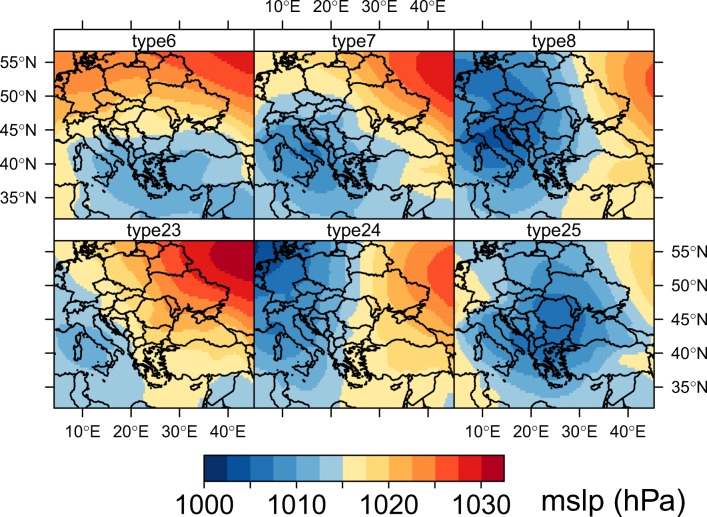
GWT circulation types resembling to Mediterranean lows upon Balkan peninsula

In order to better explain the lack of marine influences, and to track the sea salt transport and sea humidity, we analyzed the distribution of rainfall quantities (Fig. 4) upon the Balkan peninsula recorded during the occurrence of cyclonic circulations in the eastern Mediterranean basin (Fig. 5) for the 1979–2018 period.

**Fig. 6:**
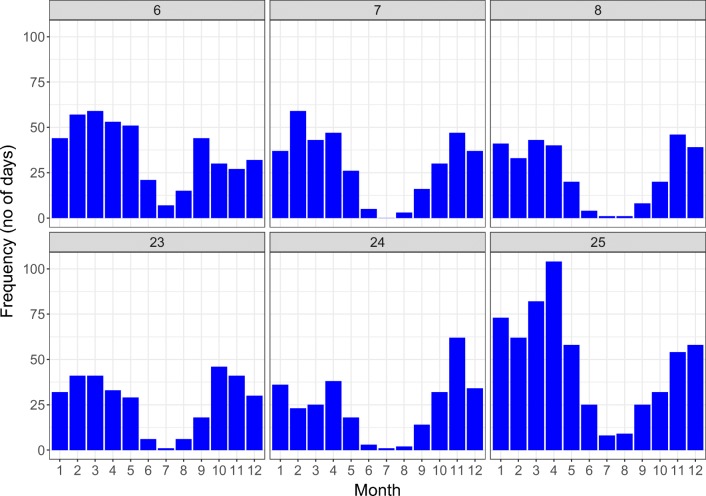
Monthly frequency of cyclonic weather types in southern Europe

Usually, these synoptic patterns consist into frontal passages moving from northern Europe towards the Mediterranean Sea. When these fronts reach southern Europe (usually the vicinity of the Gulf of Genova), they often lead to cyclogenesis, enhancing the existent baroclinic conditions. The cyclones born in this area usually follow a west to east trajectory, across the Dinaric Alps towards the Balkans inland. As they travel towards the east, their humidity content is constantly decreasing due to the orographic barrier imposed by the high elevated terrain in this area. In terms of absolute frequency, these synoptic types are more frequent in the winter, spring, and autumn and less in the summertime, when anticyclonic conditions are usually prevalent upon Europe (Fig. 6).

As a result of the orographic lifting, the sea salts concentration in the precipitation is affected due to the adiabatic processes on the windward slopes of Dinaric Alps. This also has effects upon the temperature in the air mass since the descending air on the leeward side will be warmer and subsequently drier due to the adiabatic compression.

#### Crustal enrichment factor

**Fig. 7:**
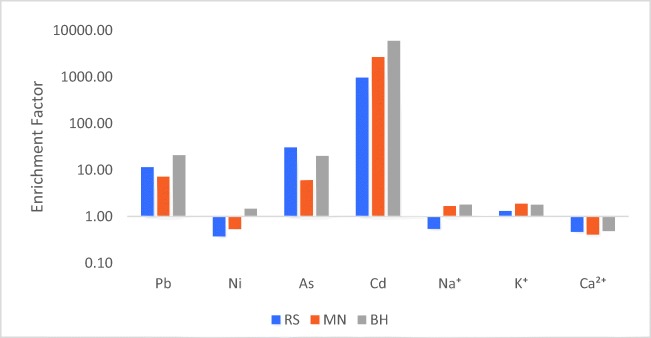
Crustal enrichment factors (EFc) for elements measured in rainwater

The assessment of crustal enrichment factor (EF_c_) represents a simple and useful way to characterize the elements in rainwater, taking under account their degree of perturbation relative to soil-originated elements (Uygur et al. 2010). In order to estimate the EF_c_ of different trace elements in rainwater, the following equation was used, considering Mg^2+^ as a reference element (Cao et al. 2009; Facchini Cerqueira et al. 2014):

10 $$ \mathrm{E}{\mathrm{F}}_{\mathrm{c}}={\left[X/{\mathrm{Mg}}^{2+}\right]}_{\mathrm{rainwater}}/{\left[X/{\mathrm{Mg}}^{2+}\right]}_{\mathrm{c}\mathrm{rust}} $$

where *X* is the concentration of the element of interest in the rainwater sample, *X*/Mg^2+^ of rainwater is the ratio from rainwater composition, and *X*/Mg^2+^ of crust represents the ratio of crustal composition (Báez et al. 2007; Barbalace 2015). Resulted values of EF_c_ are given in Fig. 7.

An EF_c_ value close to unity indicates that the only source of that element to be soil derived, while values much higher than 1 are suggesting the influence of other sources too, other than the earth’s crust (Başak and Alagha 2004). According to Poissant et al. (1994), regarding heavy metals enrichment, due to local variations in the earth’s crust composition, a limit of 10 rather than 1 is considered acceptable for crustal material. In this case, elements with an EF_c_ value between 10 and 500 are considered moderately enriched, indicating additional sources to the crustal origin, while an EF_c_ greater than 500 suggests heavily enriched state, referring to a severe contamination due to anthropogenic activities (Poissant et al. 1994).

Enrichment factors for Ca^2+^ at all sampling sites, for Ni at RS and MN for Na at RS, are < 1, suggesting that these elements have a significant crustal source, being referred to as non-enriched. Na^+^ and K^+^ were moderately enriched, while values greater than 10 for Pb and As indicated a considerable enrichment. Values for Cd exceeded the 500 limit 2 times, 4 times, and 20 times at RS, MN, and BH, respectively, indicating a severe pollution, this element being anomalously enriched and having a non-crustal source (Báez et al. 2007).

Pb and Cd originate primarily from a variety of anthropogenic sources, mining activities, and also the un-rehabilitated tailing dams. K^+^ is usually attributed to terrigenous sources, but a significant concentration of this element in the atmosphere and rainwater can also result from biomass burning and the excessive use of fertilizers. According to Uygur et al. (2010), the degree of enrichment is mainly influenced by the type, proximity, and the extent of individual sources.

#### Correlation factors

The chemical composition of rainwater is significantly influenced by the chemical composition of the atmosphere. In order to determine the relationships between ionic species, trace elements, and their provenience from different sources, Spearman’s correlation rank among the ions and heavy metals in the rainwater of the three sampling sites was calculated and is shown in Table 7. Only correlations with *R* > 0.35 were considered significant at 99% confidence level.

The conductivity is a broad indicator of the total dissolved solids in precipitation (Gioda et al. 2013), giving significant correlations with K^+^ (0.620–RS), Ca^2+^ (0.570–RS; 0.449–MN; 0.850–BH), and Mg^2+^ (0.477–RS; 0.461–MN). In the case of SO_4_^2−^, electrical conductivity is well correlated at all sampling sites, showing that probably the below-cloud scavenging mechanism takes place (Rastogi and Sarin 2005). Regarding heavy metals, electrical conductivity presents significant correlations with Pb at all sampling sites (0.42–RS; 0.45–MN; 0.56–BH), while a strong correlation between As, Ni, and Cd with electrical conductivity only exists at MN.

The significant correlation between the major exchangeable ions, Na^+^ and K^+^ (0.533–RS; 0.561–MN), may be the result of dissolution/precipitation reactions (Adams et al. 2001). The strong correlation between Ca^2+^ and Mg^2+^ at all three sampling sites (0.77–RS; 0.745–GB; 0.781–MN; 0.803–BH) implies their common origin (calcite, dolomite, and limestone dissolution, quarries, cement factories), indicating terrestrial sources (Niu et al. 2014; Xiao 2016; Rao et al. 2017). Significant correlation between Ca^2+^ and SO_4_^2−^ (0.474–RS; 0.485–MN; 0.401–BH) and between Na^+^ and SO_4_^2−^ in the case of MN (*R* = 0.582) shows the contribution of evaporitic salts, such as CaSO_4_ and Na_2_SO_4_ (Adams et al. 2001). These evaporitic salts are present in the studied area in the form of gypsum, mirabilite, and thernadite, which are signs of volcanic fumaroles and post volcanic activity but are also known as a post-mining precipitate (Stoiber and Rose 1974). Strong significant correlations can be found between SO_4_^2−^ and Pb (0.58–MN; 0.53–MN; 0.77–BH), Ni (0.63–RS; 0.78–MN and BH), and Cd (0.49–RS; 0.73–MN; 0.65–BH), which suggests the same source origin, since the studied areas are known for mining activities and the metal industry.

Positive correlations between Ca^2+^, Mg^2+^, and heavy metals can be ascribed to the composition of particulate matter, especially at MN, where the un-rehabilitated tailings dam is creating a constant problem, since thin particles of contaminated dust are carried by the wind to the atmosphere and to the neighboring shore of Serbia, leading to cross-border pollution. The lack and the weak correlations between Na^+^ and Cl^−^ at RS, BH, and MN, respectively, show that the marine influence on the precipitation chemistry is lower, and that they are emitted from other sources, such as industry or coal combustion (Ruprecht and Sigg 1990; Kaneyasu et al. 1999; Anatolaki and Tsitouridou 2009). The air masses that favor the transport of marine sprays coming from the Mediterranean and Adriatic Sea are obstructed by the Dinaric Alps and by the Balkan Mountains. As discussed earlier, the Foehn effect takes a great role in explaining the lack of correlation between sea salt ions in the rainwater. In the presence of Foehn winds, the air masses coming from the Adriatic Sea are mostly dry, having less humidity, since they are obstructed by the Dinaric Alps, leading to an orographic convection. According to Puxbaum et al. (1993), because Cl^−^ is discussed in relation to Na^+^ as sea salt aerosol, considered to be marine, it is no wonder that there are few studies that discuss the lack of correlation between sodium and chloride, and therefore other origins than marine of chloride concentration in precipitation. Due to their geographical position, since MN is situated more closely to the Adriatic and Mediterranean Sea, the higher correlation between sodium and chloride can be explained, while the insignificant correlation at RS and BH is probably due to the stronger contribution of other local sources. Since in RS a great variability of industrial activities takes place, and BH is well renowned for its mineral springs, thermal waters, and mofettas, the local emissions and strong influence of chloride concentrations can be explained.

#### Source identification using principal component analysis

Another useful tool in determining the origins of major ions in rainwater is the PCA. In the case of ionic species, a total of four factors were extracted, explaining about 77.39%, 75.17%, and 77.42% of the total variance at RS, MN, and BH, respectively. Loadings of the Varimax rotated factors for all studied areas for ionic species are presented in Table 8.

At RS, the rotated component matrix showed that Ca^2+^ and Mg^2+^ are associated in the first factor (F1), explaining 21.94% of the total variance. The main sources for Ca^2+^ and Mg^2+^ are terrestrial, resulting from the dissolution of limestones and dolomites (Rao et al. 2016; Tiwari et al. 2016; Xiao 2016). The second factor explained 19.11% of the total variance and showed high loadings of SO_4_^2−^ and Cl^−^, which can be attributed to anthropogenic sources, since the metallurgic industry which is significant in the studied area is known for coal combustion and use of chloride. F3 represented 19.07% of the total variance, having high loadings of K^+^ and NH_4_^+^, indicating the use of fertilizers and agricultural activities. The association of these two ions is a possible indicator of the use of chemical and natural fertilizers, such as NPK or animal manure. According to Behera et al. (2013), nitrogen is excreted in the form of urea (in mammals) or uric acid (in birds), which through decomposition (vaporization) can form NH_3_ and NH_4_^+^ in the atmosphere. Factor 4 has high loadings of NO_3_^−^, accounting for 17.26% of the total variance and can be attributed to traffic emissions and coal burning (Huang et al. 2008).

For MN and BH, the order of possible sources and the variance of factors are very similar. The first factor at MN and BH accounted for the 27.15% and 27.95% of the total variance, respectively, with high loadings of Ca^2+^, Mg^2+^, and SO_4_^2−^, representing mixed/crustal origins.

Primarily, Ca^2+^ and Mg^2+^ derive from terrestrial/crustal sources, through the dissolution of dolomites and limestones, but can be attributed to anthropogenic sources too, such as quarries, cement factories (Huang et al. 2008; Niu et al. 2014) and also dust from the un-rehabilitated tailing dams. SO_4_^2−^ is also an indicator of anthropogenic sources, and can originate from coal combustion, fuel burnings, and vehicle emissions but in form of post volcanic activity can be attributed to natural sources too. The second factor (F2) can be associated mainly with anthropogenic sources, representing 18.23% (MN) and 19.28 (BH) of the total variance, having high loadings of NO_3_^−^, NH_4_^+^, and K^+^. These ions can be derived from fuel combustion, coal burning, traffic emissions, animal/human excrement, and use of fertilizers, respectively (Huang et al. 2008). With 15.97% at MN and 16.86% at BH of the total variance and higher loadings of sodium and chloride, factor 3 (F3) can be considered to be of marine origin, but as the SSF showed in Table 6, the contribution of marine sources to the rainwater collected at MN and BH is not very significant, explaining also the low variance value too. F4 at MN explained 13.82% of the total variance, with high loading of K^+^, which could be attributed to the use of fertilizers and therefore agricultural sources, but is also considered as a chemical signature of biomass burning (Zunckel et al. 2003; Khare et al. 2004; Zhang et al. 2007). In the case of BH, F4 explained 12.33% having high loading of ammonium, suggesting the agricultural origin of this ion, indicating use of fertilizers and cattle waste deposits (Keresztesi et al. 2018).

Table 9 summarizes the identified dominated variables in the case of heavy metals, as well as their factor loadings and variances after using varimax rotation technique. From the principal component analysis for heavy metal concentrations, three factors were chosen that explained the 97.23%, 96.49%, and 98.27% of the total variance in the case of RS, MN, and BH, respectively. PCA in the case of heavy metals was performed in order to determine the specification within the main possible source (anthropogenic). During this analysis, natural determinative factors, as well as regional emissions, were taken under account, in order to define the possible sources. From a geological point of view, the Western and Southern Carpathians have one of the largest coal deposits from East-Central Europe, respectively, the largest metallogenic area from the Carpathians.

In the case of RS and MN, the first factor presented high loadings of Cd and Ni, with a variance of 39.28% and 44.51%, respectively. Cd and Ni are emitted by anthropogenic sources, originating from combustion under high temperature (Al-Momani 2003; García et al. 2006; Cheng and You 2010), mainly from factories and power plants which are still using coal as source of energy (Başak and Alagha 2004). Ni can also be released from oil-fired furnaces and ferroalloys (García et al. 2006). The second factor has high loadings of As at RS, as well as at MN, yielding a variance of 32.42% and 26.33%, respectively. The first factor at BH accounts for 39.94% of the total variance and has high loading values for As and Cd. As mainly comes from industrial and anthropogenic sources, but it also has a terrestrial source (Yongming et al. 2006). The most important anthropogenic inputs for As are from smelter operations and fossil fuel combustion (Smedley and Kinniburgh 2000).

At BH, F2 suggests anthropogenic sources and fossil fuel combustion, having 31.89% of the total variance and a high loading of Ni. The third factor (F3) explained the 25.53%, 25.65%, and 26.44% of the total variance having high loadings of Pb at RS, MN, and BH, respectively. F3 also represents anthropogenic sources, since Pb mainly comes from combustion processes and traffic emissions, but it also can occur from single sources, like large smelters, since a major emission source for Cd and Pb is the primary non-ferrous metal production (Szefer and Szefer 1986; García et al. 2006).

### General air flow transport patterns

The dominant trajectories and patterns were calculated using 24-h back trajectory from January 2014 to December 2017, over a 4-year period. In order to decipher the origin of the dominant air parcels first one 24-h back trajectory of every day was calculated, hence a new trajectory was started every day with the same ending height of 2000 m above ground level (AGL).

This altitude was used, since the air masses coming from south have to cross the Carpathian Mountains. The total number of back trajectories obtained was 4383, 1461 for each location. All of the back trajectories of each location were clustered using SPVAR (cluster spatial variance) calculations and then the total spatial variance (TSV) (Su et al. 2015) was calculated, respectively. SPVAR is the sum of the squared distances between the endpoints of the cluster’s component trajectories and the mean of the trajectories in that cluster (Draxler and Hess 1997; Perry and Soule 2012). Finally, the optimum number of clusters was selected using the TSV change as well.

**Fig. 8:**
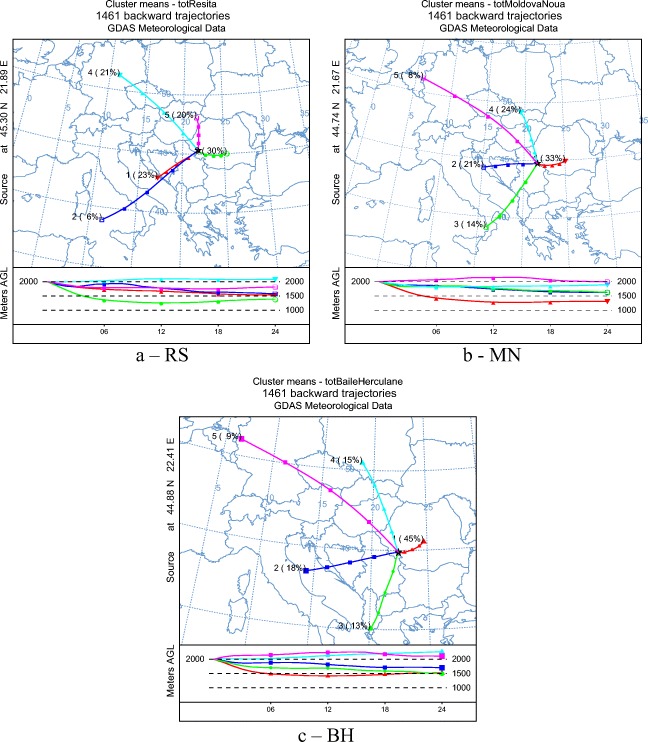
Backward trajectory distribution obtained with HYSPLIT for the three locations for the 2014–2017 period

Cluster analysis established 5 trajectory groups for each location in the studied period (January 1, 2014–December 31, 2017) (Fig. 8), which represented the general air flow pathways arriving at RS, MN, and BH in terms of direction and wind speed at 2000-m AGL. Predominant transport regimes were similar at all three locations, being classified as NW, W, SW, and S flows and regional recirculation. Analyzing Fig. 8, it can be seen that a smaller percentage of the air masses came from the NW and W flows, their sum accounting for 41% at RS, 32% at MN, and 24% in the case of BH, respectively. Air flows originating from S and SW, representing 29% at RS, 35% at MN, and 31% at BH, respectively, are also known as Foehn winds. These air masses have a traditional Foehn effect with adiabatic heating and dry-adiabatic heating from the ground surface along the fetch of the wind (Takane et al. 2017) causing sudden and dramatic increases in the temperature. One of the major impacts of this wind is desiccation that occurs in the lee of the mountain range or it may have a negative influence on the human health and behavior. The Foehn effect is responsible for the lack of correlation between sea salt ions in the rainwater collected at the studied areas. In approximately 40% of the cases at all sampling locations, air masses originating from regional recirculation (from Central and Eastern Romania) have eastern tendency, with relative short distance transport.

According to the clusters origin and transport pattern, each can be classified taking into consideration their potential atmospheric loads: anthropogenic, partially crustal impacted (clusters 1, 3, 4, and 5 for RS; clusters 1, 2, 4, and 5 for MN; clusters 1, 3, 4, and 5 for BH); and anthropogenic, marine, and partially crustal impacted (cluster 2 at RS, 3 at MN and 2 at BH, respectively). This leads to the conclusion that long-range transported aerosols and pollutants (~ 60%) originated from N, NW, and SW, while more than 30% of the aerosols (natural or anthropogenic) originated from local and nearby activities. Romania’s greatest coal mines are situated near the studied areas, while to N-NW from the sampling sites Europe’s largest ore mines can be found, with numerous tailings dams and tailings ponds. The influence of these pollutant sources can be seen in the air mass trajectory distribution as well.

## Conclusions

Rainwater composition and sources of major ions and heavy metals were studied during a period of 4 years, considering the characteristic atmospheric circulations, the main air mass transport routes, and the Foehn effect. Atmospheric circulations and distribution of precipitation amount showed the presence of the orographic convection, which affects the sea salt concentration of rainwater through the adiabatic processes that occur on the leeward slopes of the Dinaric Alps. Air masses crossing the Dinaric Alps and Balkan Peninsula lose their content of humidity through the adiabatic compression process, affecting the chemical composition of rainwater. The intensely felt effects of climate change, temperature increase, and the uneven temporal distribution of precipitation amount caused desertification in the studied area. Surface mines, tailings ponds, and dams have a significant influence on the precipitation chemistry. Trajectory cluster analysis showed that air flows originating from S and SW (influenced by Foehn) represent 29% at RS, 35% at MN, and 31% at BH of the total air flow pathways. The pH values of individual precipitation events varied between 4.33 and 8.43, with an average of 6.70. Regarding the dominance of major ions and heavy metals in rainwater, MN and BH have more resemblance, here Ca^2+^ and Mg^2+^ being the most abundant cations, SO_4_^2ˉ^ the predominant anion, and Cd the most dominant heavy metal. In the case of RS, Pb has the highest concentration, while regarding anions, the abundance of Cl^−^ can be found, followed by dominant cations like Ca^2+^, NH_4_^+^, and Mg^2+^. High concentrations of Ca^2+^ and Mg^2+^ are due to the large contribution of soil dust from the un-rehabilitated tailing dams and from the dolomite limestones. At all sampling sites, Ca^2+^ is the main neutralizing agent, but Mg^2+^ and NH_4_^+^ also participated in the neutralization process. WD fluxes for acidic species, such as SO_4_^2−^ (MN, BH) and Cl^−^ (RS), were the highest when the precipitation amounts increased, while in the case of lower precipitation amounts, alkaline species had higher WD rates. In the case of heavy metals, Cd and Pb had the highest WD fluxes, with higher concentrations measured after a period when precipitation continued for several days, while during periods with lower rain amount, the wet deposition rate was lower. The overall precipitation contamination with heavy metals at the three sampling sites was assessed by the toxicity potential and degree of contamination, which showed that precipitation at MN and BH is considerably contaminated, while at RS a moderate contamination can be observed. The values of toxicity potential suggested an elevated risk for human health in case of rainwater ingestion, especially in rural areas. Spearman correlation and PCA indicated that the chemical characteristic of the rainwater is primarily controlled by sources such as agricultural activities, mixed and crustal sources, traffic, and other anthropogenic, industrial influences, such as mining activities, smelting operations, coal combustion, and metal production. The insignificant marine influence in rainwater can be explained by the Foehn effect, characteristic to the studied region.
